# Evaluation of Citral and Green Silver Nanoparticles From *Cymbopogon citratus* Extract on Biochemical Profile and Nrf2 Gene Expression in Liver Tissue of Type 2 Diabetic Rats

**DOI:** 10.1155/bmri/9266092

**Published:** 2025-10-14

**Authors:** Shadi Khatinasab, Nasrin Kazemipour, Mohammad Foad Noorbakhsh, Saeed Nazifi, Milad Faraji, Nasrollah Ahmadi

**Affiliations:** ^1^ Department of Basic Sciences, School of Veterinary Medicine, Shiraz University, Shiraz, Iran, shirazu.ac.ir; ^2^ Department of Clinical Sciences, School of Veterinary Medicine, Shiraz University, Shiraz, Iran, shirazu.ac.ir; ^3^ Department of Pathobiology, School of Veterinary Medicine, Shiraz University, Shiraz, Iran, shirazu.ac.ir

**Keywords:** citral, diabetes, green nanoparticle, Nrf2, oxidative stress

## Abstract

The aim of this study was to evaluate the antioxidant effects of *Cymbopogon citratus*, citral, and green‐synthesized silver nanoparticles derived from the extract in mitigating oxidative stress–induced liver damage in diabetes mellitus. Seventy rats were randomly split into seven groups: a control group of healthy rats received normal saline, a cosolvent group of Type 2 diabetics received olive oil, a negative control group of Type 2 diabetics, and groups of diabetic rats receiving various treatments: metformin (100 mg/kg), *Cymbopogon citratus* extract (30 mg/kg), citral (30 mg/kg), and green‐synthesized silver nanoparticles (30 mg/kg). Type 2 diabetes was induced in rats using 90 mg/kg of nicotinamide and 65 mg/kg of streptozotocin. After 6 weeks, the rats were anesthetized to extract blood samples. The serum levels of the liver function test (serum glutamic–oxaloacetic transaminase, serum glutamic–pyruvic transaminase, and alkaline phosphatase) were measured. They were then euthanized by CO_2_ inhalation. Liver tissue was used to investigate histopathology, antioxidant activity, and Nrf2 gene expression. In the diabetes group, liver function tests increased, while TAC decreased and MDA increased (*p* < 0.05). The expression of the Nrf2 gene decreased compared to the control group (*p* < 0.05). In the treatment groups, the liver function tests and MDA decreased, while TAC and the expression of the Nrf2 gene increased compared to the Type 2 diabetes group (*p* < 0.05). The histopathological examination showed a return to normal condition. The study found that *Cymbopogon citratus*, specifically citral and green‐synthesized silver nanoparticles, effectively mitigated oxidative stress–induced liver damage in diabetes.

## 1. Introduction

With 425 million individuals affected in 2015 and over 590 million diabetics projected by 2035, diabetes mellitus is one of the fastest growing diseases in the world [1]. Diabetes is characterized by chronic hyperglycemia and impaired metabolism of carbohydrates, fats, and proteins due to defects in insulin secretion, insulin action, or both [2]. It is typically diagnosed by hyperglycemia. Hyperglycemia in diabetes produces reactive oxygen species (ROS), or free radicals, which can lead to lipid peroxidation, insulin resistance, tissue damage, and complications such as retinopathy, cardiomyopathy, neuropathy, and nephropathy [3]. Abnormally high concentrations of free radicals, combined with a decline in antioxidant defense systems, can damage cell organelles and enzymes, increase lipid peroxidation, and result in insulin resistance [4]. One of the primary organs susceptible to oxidative stress caused by hyperglycemia is the liver, which consists of insulin‐sensitive tissues [5]. Damage to liver tissue may occur due to insulin resistance in Type 2 diabetes, exacerbated by oxidative stress and abnormal inflammatory signals [6–8]. The Nrf2 signaling pathway plays a crucial role in delaying the onset of diabetes and responding to oxidative stress [9]. Due to their minimal toxicity and few side effects, herbal remedies and botanical ingredients have long been recognized as primary sources of potent antidiabetic and hypoglycemic medications [9, 10]. *Cymbopogon citratus*, or lemongrass, is a plant known for its hypoglycemic, antioxidant, anti‐inflammatory, blood pressure–lowering, and liver detoxification properties [11]. Because of its anti‐inflammatory and antioxidant qualities, citral, the primary component of the *Cymbopogon citratus* plant, serves as a supplemental treatment for diabetic patients. It influences blood serum levels of IL‐6, TNF‐*α*, haptoglobin, and *α*2 macroglobulin, among other inflammatory markers [12]. The production of these compounds as nanoparticles enhances their biological activities, prolongs their stability, inhibits their excretion, and reduces the likelihood of chemical degradation [13, 14]. Green nanoparticles exhibit a variety of therapeutic effects, including antioxidant, anticancer, and wound healing properties [15–17].

The goal of this study is to justify and investigate the therapeutic potential of citral, *Cymbopogon citratus* extract, and their green‐synthesized silver nanoparticles in mitigating diabetes‐induced oxidative stress and inflammation, with a particular focus on liver dysfunction in diabetic rats.

## 2. Materials and Methods

### 2.1. Animals

Shiraz University of Medical Sciences procured 70 healthy male Sprague Dawley rats weighing 250 ± 50 g and aged 1.5 months. Upon moving the animals to the veterinary faculty’s animal house, they were maintained at a temperature of 22°C ± 1°C, a humidity of 50%, and 12 h of light and darkness. They had free access to sanitary water and pelleted food. The study commenced 1 week after the transfer, giving the rats time to adapt to their new environment. All experimental procedures involving laboratory animals were conducted in accordance with the biological ethics regulations issued by Shiraz University’s research vice‐chancellor.

### 2.2. Aqueous Extraction Preparation

Lemongrass was collected from Fars Province, Iran, and verified by the Agricultural Faculty of Shiraz University. The leaves were washed with distilled water and air‐dried in a dark environment. Once dried, the leaves were cut into smaller pieces, mixed with distilled water in a 1:8 ratio, and boiled for 30 min. The resulting solution was filtered using Whatman No. 1 filter paper and then freeze‐dried to produce a powder.

### 2.3. Lemongrass Extract Silver Nanoparticle Synthesis

These particles were synthesized using the silver nitrate bioreduction method through *Cymbopogon citratus* extract, as per Faraji et al.’s previous study [18]. Lemongrass‐synthesized silver nanoparticles (LE‐AgNPs) were examined by DLS and transmission electron microscopy to be 68.54 and 36.24 nm, respectively. They were spherical in shape as observed by transmission electron microscopy [18].

### 2.4. Diabetes Induction

Nicotinamide (NA) (90 mg/kg, ip, Sigma, Germany) was injected, and 15 min later, animals received 65 mg/kg of streptozotocin (STZ) (ip, Sigma, Germany) intraperitoneally. After 3 days, blood glucose was measured through the tail vein. The rats were classified as diabetics if their blood glucose levels exceeded 250 mg/dL [19].

### 2.5. Experimental Design

The rats were randomly assigned to seven groups, each containing 10 individuals.

Healthy control group: No treatment intervention was implemented in this group.

Negative control group: This group received STZ along with NA as a diabetic control group. Animals in this group first received an intraperitoneal injection of 90 mg/kg of NA, followed by 65 mg/kg of STZ 15 min later. After 3 days, blood glucose was measured through the tail vein. The rats were classified as diabetics if their blood glucose levels exceeded 250 mg/dL [19].

Cosolvent control group: The diabetic rats in this group were administered 1 mL of olive oil (as a citral solvent) by gavage, starting from the beginning of the daily treatment until the study’s conclusion.

Metformin treatment: For 6 weeks, diabetic rats were administered a daily dose of 100 mg/kg BW of metformin (Sigma, Germany) in aqueous suspension via gavage [20].

Treatment 1: For 6 weeks, diabetic rats were administered a daily dose of 30 mg/kg BW of citral (Sigma, Germany) dissolved in olive oil via gavage [3].

Treatment 2: This group of diabetic animals was administered a daily oral dose of 30 mg/kg BW of *Cymbopogon citratus* extract in aqueous suspension for 6 weeks [21].

Treatment 3: Diabetic animals in this group were administered a daily dose of 30 mg/kg BW of LE‐AgNP in aqueous suspension via gavage for 6 weeks [22].

The rats were anesthetized with ketamine (100 mg/kg, ip) and xylazine (10 mg/kg, ip) for blood sampling following the conclusion of the 6‐week experiment. Following this, they were euthanized with carbon dioxide gas in the animal house of the Department of Basic Sciences at the Faculty of Veterinary Medicine, and liver tissue samples were obtained. A portion of the tissue samples was immersed in formalin for histomorphometry analysis, while another portion was stored in a −80°C freezer to assess oxidative stress and gene expression.

### 2.6. Biochemical Factor Measurement

A 5‐cc syringe was employed to collect blood serum from the heart. Blood serum was extracted by centrifuging the blood sample at 3000 rpm for 10 min. After that, the blood serum was kept in a freezer at −20°C for a week to conduct further examination. Then, blood serum was tested for biochemical markers of liver function, such as alkaline phosphatase (ALP), serum glutamic–pyruvic transaminase (SGPT), and serum glutamic–oxaloacetic transaminase (SGOT), using commercial kits (Pars Azmoon Co., Tehran, Iran) and a biochemical auto‐analyzer (Alpha Classic AT++, Sanjesh, Iran).

### 2.7. Histopathological Investigations

The liver tissue sample was immersed in a 10% formalin solution for histopathological analysis. Through a standard procedure, tissue sections were made from samples fixed in 10% formalin for microscope examination. The hematoxylin–eosin (H&E) method was then employed to stain the sections. The histological structure of the liver was analyzed and compared in various groups using an optical microscope (Olympus E450, Japan).

### 2.8. Antioxidant Capacity Evaluation

The initial step in homogenizing liver tissue was to remove 100 mg using a digital scale and freeze it with liquid nitrogen. Subsequently, the tissue was homogenized in a mortar with 1 mL of phosphate‐buffered saline (PBS). It is essential to mention that all procedures were executed on ice. The homogenized tissue was put into a microtube and centrifuged for 10 min at 6000 rpm in a refrigerator‐cooled centrifuge at 6°C. Finally, the supernatant was collected. The malondialdehyde (MDA) and total antioxidant capacity (TAC) levels were then measured by a commercial kit (Zelbio Co., Germany) in the supernatant.

### 2.9. Measurement of Nrf2 Gene Expression

Total RNA was extracted from liver tissues using the FavorPrep Tissue Total RNA Mini Kit (FAVORGEN Biotech Corporation, Taiwan). To assess the quality of the extracted RNA, 5 *μ*L was electrophoresed on an agarose gel, and its absorbance was measured at 260 nm using a Thermo Scientific NanoDrop Lite Spectrophotometer. Purity was confirmed with a ratio of 1.8–2.0 at A260/A280. Subsequently, the total RNA was converted into cDNA using the RevertAid First Strand cDNA Synthesis Kit (Thermo Scientific, Waltham, Massachusetts, United States) and analyzed using a StepOnePlus Real‐Time PCR System (Applied Biosystems). All primers were designed using Allele IDv7.8 software. The following primers were employed: For Nrf2, the forward primer is 5 ^′^‐CACATCCAGACAGACACCAGTC‐3 ^′^, and the reverse primer is 5 ^′^‐CTACAAATGGGAATGTCTCTGC‐3 ^′^. The TBP (TATA‐binding protein) transcript was used as an internal control, with the forward primer 5 ^′^‐GCGGGGTCATGAAATCCAGT‐3 ^′^ and the reverse primer 5 ^′^‐AGTGATGTGGGGACAAAACGA‐3 ^′^. The quantities of the target and housekeeping genes (Nrf2 and TBP) were determined using a comparative cycle threshold (Ct) method. The gene expression level was determined using the 2^−*ΔΔ*Ct^ formula.

### 2.10. Data Statistical Analysis

The mean ± standard error of the mean (SEM) was used to report the data. The ANOVA statistical test, Tukey’s HSD post hoc test, and GraphPad Prism Version 10.1 software were used to analyze the research data statistically. With an accuracy of *p* ≤ 0.05, a statistical difference was deemed significant.

## 3. Results

### 3.1. Liver Function Serum Biochemical Parameters

#### 3.1.1. Measurement of SGOT Activity

**Figure 1:**
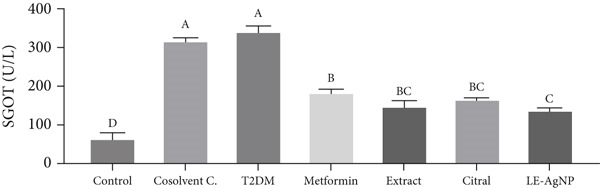
The influence of *Cymbopogon citratus* extract, citral, and LE‐AgNP on serum SGOT (*n* = 10). Results are presented as mean ± SEM and compared using the ANOVA and Tukey post hoc tests. Significant differences (*p* < 0.05) are indicated by different lowercase letters. SGOT: serum glutamic–oxaloacetic transaminase, T2DM: Type 2 diabetes mellitus, and LE‐AgNP: lemongrass‐synthesized silver nanoparticle. Different letters in the columns indicate significant differences.

The Type 2 diabetes group had serum levels of SGOT of 344.1 ± 13.09 U/L, which were significantly higher than those of the control group (67.87 ± 11.95 U/L) (*p* < 0.0001). SGOT concentrations were 186.9 ± 5.59, 150.3 ± 12.81, 169.0 ± 1.73, and 141.6 ± 3.48 U/L in the metformin, *Cymbopogon citratus* extract, citral, and LE‐AgNP treatment groups, respectively. They decreased significantly compared to the Type 2 diabetes group (*p* < 0.0001) and were comparable to the control group. The nanoparticle treatment group exhibited the lowest level of blood SGOT among the treatment groups; however, no significant difference was observed between the treatment groups (*p* > 0.05) (Figure 1).

#### 3.1.2. Measurement of SGPT Activity

**Figure 2:**
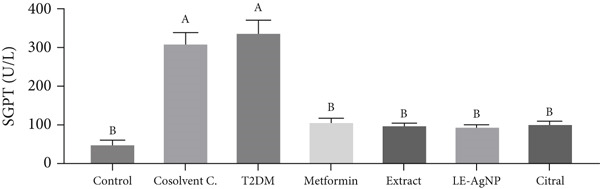
The influence of *Cymbopogon citratus* extract, citral, and LE‐AgNP on serum SGPT (*n* = 10). Results are presented as mean ± SEM and compared using the ANOVA and Tukey post hoc tests. Significant differences (*p* < 0.05) are indicated by different lowercase letters. SGPT: serum glutamic–pyruvic transaminase, T2DM: Type 2 diabetes mellitus, and LE‐AgNP: lemongrass‐synthesized silver nanoparticle. Different letters in the columns indicate significant differences.

The serum SGPT level of the Type 2 diabetic group was 340.3 ± 29.83 U/L, which was significantly higher than that of the control group (52.92 ± 6.46 U/L) (*p* < 0.0001). The SGPT concentrations in the groups treated with metformin, *Cymbopogon citratus* extract, citral, and LE‐AgNP were 109.2 ± 6.46, 101.2 ± 2.44, 104.0 ± 5.56, and 98.35 ± 1.62 U/L, respectively. The results indicated that they were significantly lower than those of the Type 2 diabetes group (*p* < 0.0001) and were comparable to those of the control group. The nanoparticle treatment group exhibited the lowest level of blood SGPT among the treatment groups; however, no significant difference was observed between the treatment groups (*p* > 0.05) (Figure 2).

#### 3.1.3. Serum ALP Activity

**Figure 3:**
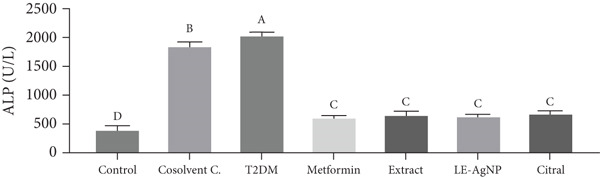
The influence of *Cymbopogon citratus* extract, citral, and LE‐AgNP on serum ALP (*n* = 10). Results are presented as mean ± SEM and compared using the ANOVA and Tukey post hoc tests. Significant differences (*p* < 0.05) are indicated by different lowercase letters. ALP: alkaline phosphatase, T2DM: Type 2 diabetes mellitus, and LE‐AgNP: lemongrass‐synthesized silver nanoparticle. Different letters in the columns indicate significant differences.

The ALP serum level of the Type 2 diabetic group increased significantly by 395% compared to the control group (*p* < 0.0001). Compared to the Type 2 diabetes group, the ALP concentrations in the metformin, *Cymbopogon citratus* extract, citral, and LE‐AgNP treatment groups were significantly reduced by 69%, 66%, 67%, and 69%, respectively (*p* < 0.0001), and were comparable to the control group. The metformin treatment group exhibited the lowest blood ALP level among the treatment groups; however, no statistically significant difference was observed between the treatment groups (*p* > 0.05) (Figure 3).

### 3.2. Determining the Antioxidant Activity of Liver Tissue

#### 3.2.1. Antioxidant Parameters in Liver Tissue

##### 3.2.1.1. TAC

The tissue TAC level in the Type 2 diabetic group was 26.48 ± 0.57 * μ*M/100 mg tissue, indicating a significant reduction in comparison to the control group (57.99 ± 2.56 * μ*M/100 mg tissue) (*p* < 0.0001). TAC concentrations were 35.94 ± 1.18, 41.07 ± 0.65, 36.02 ± 1.00, and 28.47 ± 1.55 * μ*M/100 mg tissue in the metformin, *Cymbopogon citratus* extract, citral, and LE‐AgNP treatment groups, respectively. They significantly increased and approached the control group compared to the group with Type 2 diabetes. The LE‐AgNP treatment group exhibited the highest levels of tissue TAC among the treatment groups; however, no significant difference was observed between the treatment groups (*p* > 0.05) (Figure 4a).

Figure 4The effects of *Cymbopogon citratus* extract, citral, and LE‐AgNP (*n* = 5) on (a) TAC and (b) MDA in liver tissue. The results are presented as mean ± SEM and compared using the ANOVA and Tukey’s post hoc tests. Different lowercase letters denote statistically significant differences (*p* < 0.05). TAC: total antioxidant capacity, MDA: malondialdehyde, T2DM: Type 2 diabetes mellitus; LE‐AgNP: lemongrass‐synthesized silver nanoparticle. Different letters in the columns indicate significant differences.

**Figure:**
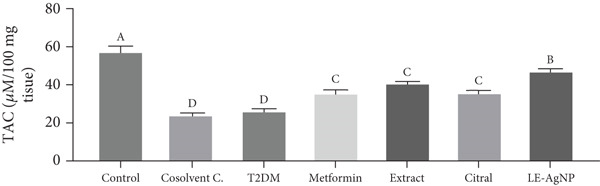
(a)

**Figure:**
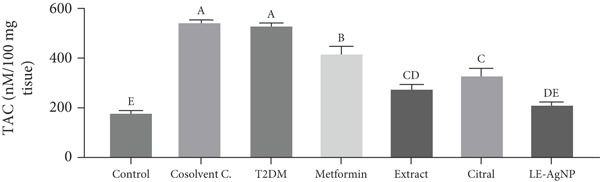
(b)

##### 3.2.1.2. MDA

Compared to the control group (184.8 ± 6.24 nM/100 mg tissue), the tissue MDA levels in the Type 2 diabetic group were significantly higher at 542.6 ± 3.98 24 nM/100 mg tissue (*p* < 0.0001). MDA concentration values were 424.4 ± 23.07, 281.9 ± 15.32, 335.9 ± 25.03, and 218.9 ± 6.19 24 nM/100 mg tissue in the metformin, citral, *Cymbopogon citratus* extract, and LE‐AgNP, respectively. Compared to the Type 2 diabetes group, they experienced a significant decrease (*p* < 0.0001) and converged with the control group. The LE‐AgNP treatment group experienced the lowest tissue MDA level among the treatment groups; however, no statistically significant difference was observed between the treatment groups (*p* > 0.05) (Figure 4b).

### 3.3. Nrf2 Gene Expression in Liver Tissue

**Figure 5:**
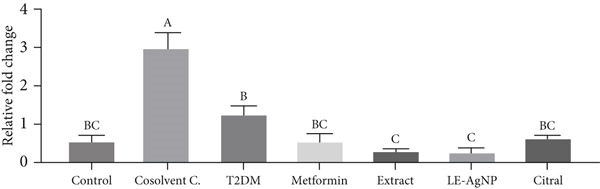
The effects of *Cymbopogon citratus* extract, citral, and LE‐AgNP (*n* = 5) on Nrf2 gene expression in liver tissue. The results are presented as mean ± SEM and compared using the ANOVA and Tukey’s post hoc tests. Different lowercase letters denote statistically significant differences (*p* < 0.05). T2DM: Type 2 diabetes mellitus; LE‐AgNP: lemongrass‐synthesized silver nanoparticle. Different letters in the columns indicate significant differences.

The Type 2 diabetes group had a tissue Nrf2 gene expression level of 0.58 ± 0.14. A significant decrease was observed in contrast to the control group (0.1 ± 0.31). The Nrf2 gene expression levels were 1.14 ± 0.16, 1.15 ± 0.25, 1.29 ± 0.27, and 1.04 ± 0.20 in the metformin, *Cymbopogon citratus* extract, citral, and LE‐AgNP treatment groups, respectively. Compared to the Type 2 diabetes group, they increased, but the difference was not statistically significant (*p* > 0.05) and was comparable to the control group. Although the Nrf2 gene expression associated with the LE‐AgNP was more similar to that of the control group in the treatment groups, no significant difference was observed between the treatment groups (*p* > 0.05) (Figure 5).

### 3.4. Liver Tissue Histopathological Results

#### 3.4.1. Healthy Control Group

**Figure 6:**
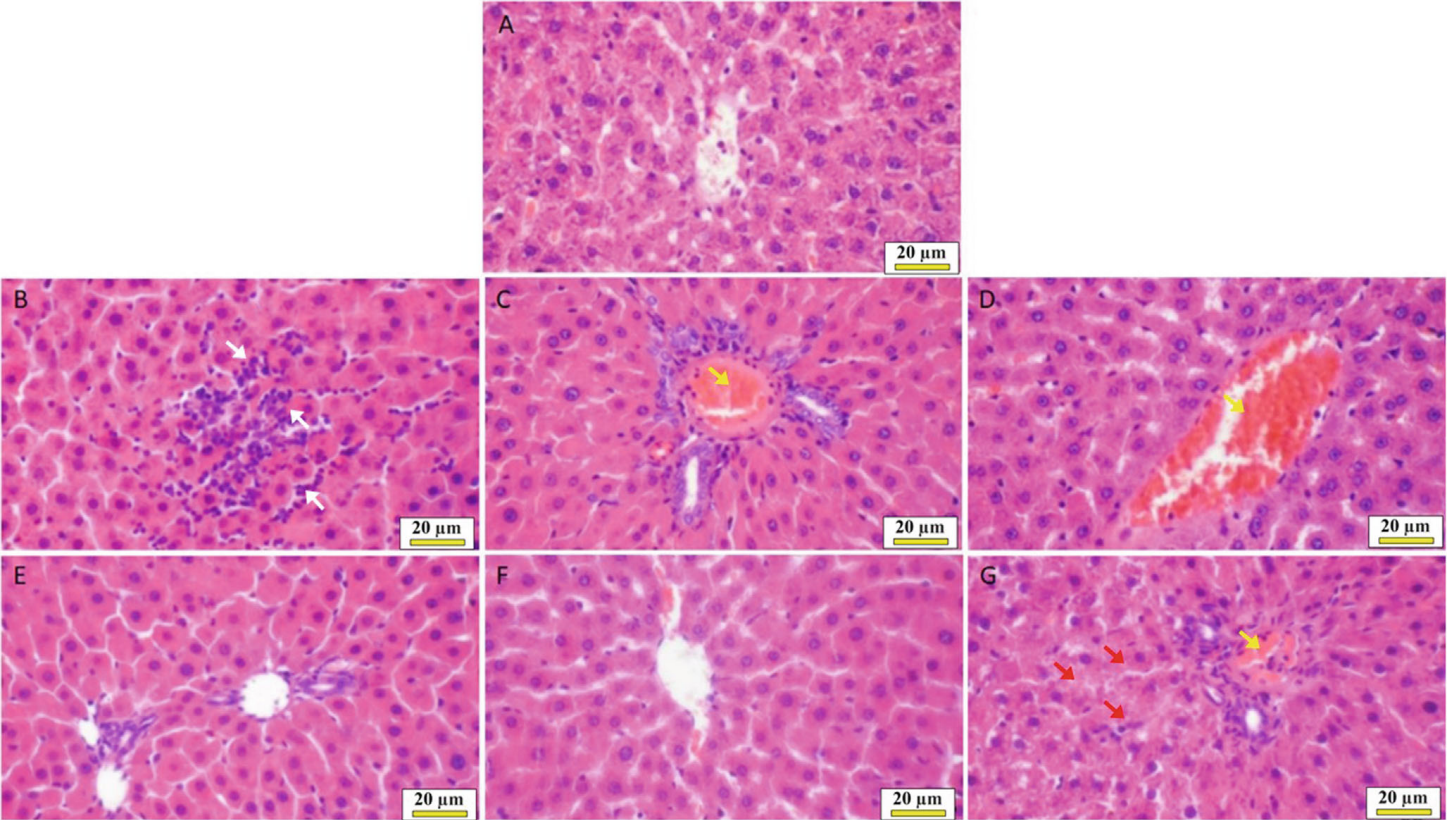
The effects of *Cymbopogon citratus* extract, citral, and LE‐AgNP (*n* = 5) on the histopathology of liver tissue: (A) control group, (B) solvent group, (C) negative control group Type 2 of diabetes, (D) metformin, (E) *Cymbopogon citratus*, (F) citral, and (G) LE‐AgNP (H&E, ×400). Mononuclear inflammatory cells (), congestion (), and granular background changes ().

Within the typical structure of the histological architecture, liver cells in the form of cell plates with a thickness of one or two cells, separated by sinusoids, were present in the histological sections of rats’ livers from the healthy control group. No signs of necrosis or cellular changes suggested pathological lesions, such as the development of cytoplasmic vacuoles or cell death. No inflammatory cell accumulation was seen in the liver parenchyma, including neutrophils or lymphocytes, hemorrhage, fibrosis, or vascular dilatation with sinusoids (Figure 6A).

#### 3.4.2. Solvent Group

The histopathological examination of the liver tissue sections associated with the solvent group revealed the accumulation of mononuclear inflammatory cells and focal necrosis of liver cells. However, the liver parenchyma exhibited no fibrosis or hemorrhage (Figure 6B).

#### 3.4.3. Type 2 Diabetes Group

In this group of rats, the cytoplasm of the liver cell tissue section exhibited severe hyperemia of the liver vessels and background granular changes (Figure 6C).

#### 3.4.4. Metformin Treatment Group

The rats in this group exhibited hyperemia in their histological liver sections. Within the lobular structure of the liver, one or two fully functional nuclei, shaped like cell plates, can be found radially around the central vein and in the center of the cell. The cytoplasmic background changes, and the foci showing the presence of inflammatory cells were not observed (Figure 6D).

#### 3.4.5. *Cymbopogon citratus* Extract Treatment Group

The liver cells of the rats in the essential oil treatment group exhibited uniform eosinophilic cytoplasm, with one or two complete healthy nuclei located in the center of the cell. They are arranged radially around the central vein of the liver’s lobular structure in cell plates, each with a thickness of one or two cells, and are divided by sinusoids. There was no evidence of cell swelling, degeneration or vacuole formation, necrosis, cell death, or an increase in fibrous connective tissue, inflammatory cells, or vascular lesions in the liver tissue sections associated with this group (Figure 6E).

#### 3.4.6. Citral Treatment Group

Pathological changes, including cell necrosis, the presence of inflammatory cells, and vascular lesions such as hyperemia, hemorrhage, and fibrosis, were not observed in the liver parenchyma during the histopathological examinations of the tissue sections of this group (Figure 6F).

#### 3.4.7. Nanoparticle‐Treated Group

The histological sections of the liver associated with this group did not exhibit any pathological changes, including hepatocellular necrosis, infiltration of inflammatory cells, hemorrhage, or fibrosis in the liver parenchyma. However, the liver tissue sections of the rats in this group exhibited signs of vascular hyperemia (Figure 6G).

## 4. Discussion

Type 2 diabetes mellitus (T2DM) is one of the most rapidly expanding chronic diseases globally, with projections estimating it will affect 693 million adults by 2045. It results from insulin resistance and/or inadequate insulin secretion and leads to persistent hyperglycemia, a condition that triggers oxidative stress through mechanisms such as excessive mitochondrial ROS generation and NADPH oxidase activation [23, 24]. This oxidative stress is a critical factor contributing to complications, including liver injury [25].

The liver plays a central role in glucose homeostasis, and oxidative stress directly affects its function. In the current study, rats with T2DM exhibited elevated MDA levels and decreased TAC, confirming the presence of oxidative stress. Concurrently, increased serum levels of liver enzymes—ALP, SGPT, and SGOT—indicated hepatic injury. These findings align with prior studies showing that oxidative stress in diabetes impairs liver function and leads to hepatocellular damage [26–28].

Histopathological analyses of diabetic liver tissue confirmed liver damage, showing hyperemia, necrosis, and inflammatory infiltration. These results were consistent with those reported by Aghemo et al. and Ferro et al., where chronic oxidative stress led to liver inflammation, hepatic stellate cell activation, and fibrosis [29, 30]. Naseri et al. further correlated elevated liver enzymes and oxidative markers in liver‐injured diabetic rats [31]. Similarly, Balamash et al. confirmed that elevated ALP, SGOT, and SGPT levels in diabetic rats are associated with liver apoptosis and necrosis [32].

The antioxidant defense system, including enzymes like superoxide dismutase (SOD), catalase (CAT), and glutathione peroxidase, plays a crucial role in neutralizing ROS. Nrf2, a transcription factor, regulates these antioxidant genes. However, hyperglycemia suppresses Nrf2 activity, reducing antioxidant defenses and exacerbating oxidative stress [33–36]. In the present study, Nrf2 gene expression was reduced in diabetic rats, confirming its suppression in diabetes‐induced oxidative conditions.

Metformin, the frontline therapy for T2DM, primarily lowers blood glucose by inhibiting hepatic gluconeogenesis and glycogenolysis [37, 38]. Additionally, it has been reported to enhance antioxidant defenses. Studies by Balamash et al., Wang et al., and Yasmin et al. confirmed metformin’s ability to restore antioxidant enzyme levels and reduce liver enzyme markers [32, 39, 40]. However, while metformin demonstrated efficacy in reducing oxidative stress and liver enzyme levels, its effects were found to be less pronounced compared to natural antioxidant treatments in this study.

Antioxidants from natural sources—such as vitamins E and C, alpha‐lipoic acid, and selenium—have been shown to ameliorate oxidative stress in diabetes [41–43]. In particular, plant‐based antioxidants hold therapeutic promise. *Cymbopogon citratus* (lemongrass) is rich in flavonoids and phenolic compounds, contributing to its potent antioxidant activity. Essential oils and bioactive compounds from this plant, especially citral, have shown hypoglycemic and hepatoprotective effects [44, 45].

In this study, *Cymbopogon citratus* extract, its major compound citral, and its green‐synthesized silver nanoparticles were used to treat diabetic rats. The treatment groups showed reduced MDA and elevated TAC levels, indicating alleviation of oxidative stress. Biochemical markers (ALP, SGOT, and SGPT) were also significantly lowered, suggesting hepatic protection. These findings are supported by previous studies: Guleria and Sehgal demonstrated lemongrass’s ability to inhibit lipid peroxidation and scavenge free radicals [46]. Somparn et al. showed that lemongrass extract improves antioxidant enzyme levels and decreases liver enzyme markers [47]. Saenthaweesuk et al. reported similar results, showing that the extract mitigates paracetamol‐induced liver injury via antioxidant action [48].

Citral, the main component of *Cymbopogon citratus*, has also been validated for its hepatoprotective effects. Júnior et al. reported decreased SGPT and SGOT levels in diabetic rats treated with citral, indicating reduced oxidative liver injury [49]. Akinbosola et al. observed that both citral and lemongrass oil increased liver antioxidant enzymes (SOD and CAT) and reduced liver enzyme markers, emphasizing their protective effects [50].

Furthermore, the green synthesis of silver nanoparticles using plant extracts has emerged as a promising, eco‐friendly, and effective therapeutic approach. These nanoparticles exhibit enhanced biological activity due to their increased surface area [51]. Al‐Salmi et al. demonstrated the superior antioxidant effect of green‐synthesized nanoparticles compared to green tea extract alone, as indicated by normalized MDA levels [52]. In the present study, nanoparticles synthesized from *Cymbopogon citratus* exhibited the most potent therapeutic effect among the tested treatments, reducing MDA and liver enzyme markers more effectively than the extract or citral alone.

Hammam et al. confirmed the safety and antioxidant efficacy of green‐synthesized nanoparticles from *Cymbopogon citratus* essential oil. They found that these nanoparticles reduced serum MDA and liver enzyme levels without inducing cytotoxicity [53]. Similarly, Rahimi et al. and Rajappa et al. demonstrated that natural antioxidants and their nanoparticle forms activate Nrf2 gene expression more effectively than conventional drugs, providing better protection against oxidative damage [54–56].

This study also found that treatment with *Cymbopogon citratus*, citral, and their green‐synthesized nanoparticles increased Nrf2 gene expression, suggesting that their antioxidant properties may be mediated via activation of this pathway. This is consistent with findings by Gandhi, who highlighted the potential of citral in modulating the Nrf2 signaling pathway in diabetes [57].

A comparative analysis of all treatments revealed that green‐synthesized nanoparticles provided the most potent hepatoprotective and antioxidant effects, followed by *Cymbopogon citratus* extract, citral, and, lastly, metformin. These findings suggest that while metformin remains a valuable antidiabetic drug, plant‐derived compounds—particularly in nanoparticle form—may offer enhanced therapeutic effects by targeting oxidative stress and protecting hepatic tissues.

Despite these promising results, several limitations must be acknowledged. The current study was conducted on rats, and while they are widely accepted as a model for diabetes research, the findings cannot be directly extrapolated to humans. Additionally, the long‐term effects and safety of silver nanoparticles in biological systems remain underexplored. Hence, further clinical studies and long‐term toxicological assessments are needed.

Oxidative stress plays a pivotal role in the pathogenesis of T2DM and its hepatic complications. *Cymbopogon citratus*, citral, and their green‐synthesized nanoparticles offer significant antioxidant and hepatoprotective effects by reducing oxidative biomarkers, normalizing liver enzymes, and upregulating Nrf2 expression. These natural compounds, especially in nanoparticle form, have the potential to complement or even surpass standard pharmacotherapy in managing diabetes‐induced hepatic injury. This study used only a rat model, limiting human applicability. Short‐term treatment and a narrow focus on markers restrict understanding of long‐term effects and broader mechanisms. Molecular and histological analyses were limited, and glycemic control was not assessed. Future studies should expand these aspects and include dose–response evaluations.

## 5. Conclusion

This study shows that diabetes causes liver oxidative stress, marked by increased MDA, decreased TAC, elevated liver enzymes, and lowered Nrf2 expression. Treatments with *Cymbopogon citratus* extract, citral, and their green‐synthesized silver nanoparticles (LE‐AgNPs) reduced oxidative stress, improved antioxidant levels, normalized liver enzymes, and boosted Nrf2 expression. These findings highlight their potential as natural, complementary therapies for protecting liver function in diabetes.

## Conflicts of Interest

The authors declare no conflicts of interest.

## Funding

This study was supported by Shiraz University. No additional external funding was received for this work.

## Data Availability

The datasets generated and/or analyzed during the current study are available from the corresponding author on reasonable request.
